# Biophysical methods in early drug discovery

**DOI:** 10.5599/admet.733

**Published:** 2019-12-11

**Authors:** Geoffrey Holdgate, Kevin Embrey, Alexander Milbradt, Gareth Davies

**Affiliations:** 1 Hit Discovery, Discovery Sciences, R&D, AstraZeneca, Alderley Park, UK; 2 Structure, Biophysics and FBLG, Discovery Sciences, R&D, AstraZeneca, Cambridge, UK

**Keywords:** Affinity selection mass spectrometry, surface plasmon resonance, nuclear magnetic resonance, differential scanning fluorimetry, isothermal titration calorimetry, microscale thermophoresis

## Abstract

Biophysical methods such as mass spectrometry, surface plasmon resonance, nuclear magnetic resonance, and both differential scanning isothermal titration calorimetry are now well established as key components of the early drug discovery process. These approaches are used successfully for a range of activities, including assay development, primary screening, hit confirmation and detailed mechanistic characterisation of compound binding. Matching the speed, sensitivity and information content of the various techniques to the generation of critical data and information required at each phase of the drug discovery process has been key. This review describes the framework by which these methods have been applied in the drug discovery process and provides case studies to exemplify the impact.

## Introduction

Biophysical methods, which can encompass a wide range of techniques focussed on measuring the structure, properties, dynamics or function of biomolecules, have been increasingly employed in the drug discovery process since their first introduction in the early 1990s. It was during this time that commercial instruments such as the first isothermal titration calorimeter (ITC) [1] and the first surface plasmon resonance (SPR) [2] instruments became available and their use exemplified using biochemical systems. Alongside this, existing biophysical approaches were used in novel ways to identify and characterise protein-ligand interactions, for example the first report of the use of affinity selection, coupled to detection by mass spectrometry, for the identification of molecules binding to a macromolecule [3], and the use of nuclear magnetic resonance (NMR) to identify fragments that could subsequently be optimised and linked to form more potent compounds [4]. The development of these biophysical methods coincided with the advent of high-throughput screening (HTS), leading from natural product screening of a few hundred compounds each week in the late 1980s, through to HTS hits being responsible for starting matter for almost half of drug companies’ portfolios in the mid-1990s [5,6]. This allowed the valuable combination of biophysics with HTS to contribute to the establishment of high quality, high-throughput assays through the characterisation of protein and tool ligands, as well as the evaluation of HTS output through orthogonal application of biophysical methods to screen for true target engagement [7]. More recently, biophysical methods have matured, throughput has increased, and sensitivity improved, such that some of these methods can also now be employed in primary screening, not only for fragments, which typically screen lower numbers of low molecular weight compounds [8], but for screening libraries comprising many thousands of compounds [9]. The high-fidelity nature of many biophysical methods, coupled to the specific information content they can access, has also meant that they are used increasingly to characterise the mode of action of hits and leads. Some methods provide access to kinetic data [10], whilst others enable thermodynamic characterisation of ligand binding [11]. Others may allow structural insights into binding mode and binding site [12,13]. Our understanding of the strengths and weaknesses of the methods, as well as a better appreciation of the impact that results from appropriate positioning within the drug discovery process has contributed significantly to the increased use of biophysical methods observed today. This review will provide an overview of the benefit that can be gained from the incorporation of biophysical methods within areas of the drug discovery process and will provide case studies to exemplify their impact.

## Assay development

The capability to design, build and implement assays that are specific, robust and sensitive enough to identify and characterise potential new drug molecules is fundamental to drug discovery. In developing a new assay there are several factors that must be considered. These include: the nature of the reagents, such as their identity, purity, concentration, functionality and stability; the features of the detection system, such as the sensitivity, dynamic range, potential for interference and reproducibility; the analysis of the data; and the subsequent statistical examination of assay performance. The application of biophysical methods early during assay development can help to understand some of these features and to ensure that high-fidelity, fit for purpose assays are developed. Of course, this extra resource needs to be considered during project planning, but the benefits of applying this early outweighs the resource that would need to be applied to rescue projects which have been misled or have failed due to spurious activity from poorly characterised reagents or assays.

### Reagent quality control

Early application of biophysical methods often focusses on understanding the quality of the reagents available, both in terms of the suitability of the target protein and of the behaviour of the known tool compounds. For a protein to be deemed suitable to be used in subsequent drug discovery assays it should fit criteria associated with the group of characteristics highlighted in the paragraph above. The protein should be the right protein, so its identity should be confirmed, otherwise invalid and misleading results may be obtained. The purity of the protein is also important as impurities may have similar activities or bind test compounds. The concentration of the protein should be measured so that considerations around concentration dependent effects can be understood. The functionality of the protein should also be investigated. This may require measurements of ligand binding, the catalytic activity (in the case of enzymes) or the ability to carry out other functions or interactions. Finally, the stability of the protein should be assessed, both in terms of its thermodynamic and kinetic stability, as well as its ability to withstand certain conditions such as freeze-thawing. It also may be necessary to characterise the binding of other molecules such as substrates, cofactors, binding partners or competing probe molecules, to understand their requirement and concentration range desired and permitted within the assay methodologies under consideration. These attributes of the target protein may be investigated using a variety of approaches and biophysical methods can help to provide confidence in the target protein, by being employed alongside or instead of standard biochemical methods for protein characterisation. Table 1 shows several possible methods that may be employed in such characterisation and it highlights the information that is generated to allow effective decision making to ensure that only protein of sufficient quality is used in hit-finding and subsequent optimisation assays. Some of these methods may also be used to characterise the binding of ligands to the target protein, to evaluate their purity and concentration.

Clearly, it is essential to understand the quality of the protein and its behaviour before significant time is spent developing assays and certainly before bulk amounts of protein are made for activities such as high-throughput screening and X-ray crystallography. In this respect it is useful to remember that the target protein goes into every well of the experiment, and so issues with the performance of this reagent has the potential to compromise the entire experiment. This can be contrasted with efforts to ensure individual compound purity and activity. Although individual compound integrity is important to ensure that the identification of useful chemical start points from any active hit compound is not missed, it does not have the same propensity to compromise the whole hit identification process, as would be the case for a protein of questionable integrity.

Protein integrity, and its resulting ability to bind to test compounds which may then modulate the biological function, can be compromised in several ways, some examples of which are illustrated in Table 2.

The application of biophysics to characterise reagent quality may not be a large, resource-intensive effort. Often, a single but decisive experiment can be extremely informative, and often critical in understanding the behaviour of the target protein. Additionally, since these methods are often relatively generic, requiring little assay development, their use at this early stage is not prohibitive, and is often influential and impactful. Even if methods do require additional development time, for example SPR assays may take longer to establish than a simple ITC experiment, this is usually time well invested, as the methods are frequently used again for hit evaluation, and so the development time is in effect just positioned earlier in the workflow than it otherwise may have been. In the case of SPR, having an assay in place to characterise hits post HTS is very valuable, and will be discussed further below.

### Assay quality control

In addition to being able to characterise reagents, biophysical methods are also an invaluable tool applied to evaluating assays for their ability to identify compounds that engage with the target protein to bring about the desired effect. Any biochemical assay has the potential for artefacts to arise due to non-desired mechanisms. These may be specific mechanisms that are unwanted in a drug-like compound, such as reactivity (for example thiol reactivity), redox cycling, colloidal aggregation, heavy metal contamination, protein unfolding, protein denaturation (so called pan assay interference compounds or PAINs [14]) or biological system or technology interferences, such as coupled enzyme inhibitors, fluorescent compounds or quenchers. Understanding the liability of a biochemical assay to these types of compounds helps to understand the potential output from high-throughput screens (HTS) utilising that assay approach. At AstraZeneca, HTS development includes testing a small library of around 1000 compounds with unwanted mechanism of inhibition (the uMOI set) and around 7000 compounds (the validation set), which are meant to represent the diversity present in the full screening set of around 2 million compounds. This allows the assay to be assessed in terms of its susceptibility to PAINs, as well as providing an assessment of reproducibility, likely hit rate and to highlight potential artefacts and propensity for false positives and negatives. Biophysical methods such as SPR or NMR are employed following this early screening activity to characterise the hits, so that the knowledge arising from an understanding of the reasons behind false positives may be used to further optimise the assay to avoid these types of hits in the full screen. Table 3 exemplifies several assays, across different target classes, where biophysical characterisation post validation set testing, influenced the subsequent hit identification strategy or tactics.

**Figure 1.**
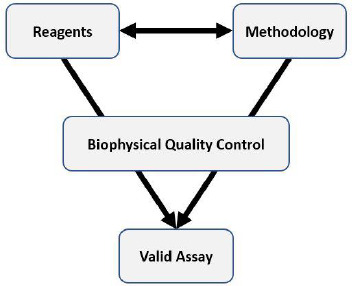
Interplay between reagents, assay methodology and biophysical quality control in the development of valid assays.

This workflow therefore allows decisions to be made based on any specific issues that arise due to the nature of the assay, that may be mitigated or avoided by modifying the screening cascade. For example, knowing that heavy metals contaminants or redox cycling compounds may be hits in the biochemical assay, but the compounds are not true binders to the target protein in a high-fidelity biophysical method allows the primary screen to be modified to reduce the liability to such effects. In these examples, the use of metal chelating agents and investigating different reducing agents may lead to changes to the assay protocol. Alternatively, post screen triage may involve introducing additional assays that allow compounds functioning by these undesirable mechanisms to be identified and deprioritised. Thus, the use of biophysical methods in this way represents a valuable investment to increase the probability that high-quality hits will be identified during the primary screen. As can be seen from Table 3, in some cases, the impact of the biophysical testing was taking a decision not to run the primary screen at all, but to pursue alternative approaches, such as fragment-based lead generation (FBLG). These decisions, although difficult at the time, due to the previously committed investment, ultimately may result in substantial cost savings, firstly from not committing to HTS (at an average cost of around $90k) and secondly from not following up spurious hits. Figure 1 shows the interplay that is required between the assay methodology, the use of high-quality reagents and the role that biophysics has in helping to characterise these aspects to facilitate the implementation of valid screening assays.

## Primary screening

There are few biophysical methods that can be applied to primary screening as usually defined in the high-throughput setting, which often refers to the testing of 1 million or more compounds, and usually these methods are more frequently applied for secondary hit evaluation. The predominate reasons are the amount of protein required and the throughput required to achieve primary screening in a reasonable timeframe. To address these issues compound mixtures are often used to facilitate reductions in reagents and time, since many compounds may be screened from a single well. Often, mixtures have no adverse effects on the protein, or on the ability to detect binding, but sometimes problems are experienced if there are compound-compound interactions or if the compound organic load or the compound solvent concentration [DMSO] is too high.

Two biophysical methods that are often capable of providing throughputs approaching that of traditional HTS are affinity selection mass spectrometry (AS-MS) [15] and thermal shift assays (TSA) [16]. These methods utilise mass detection of ligands bound to a target protein and the ligand-induced increase in thermal stability respectively to identify hits. The benefit of these approaches in primary screening is that the assay is generic and can rapidly be optimised for the target of interest. The disadvantage in biophysical primary screening is that identified hits may bind to the target protein but may not have the desired biological effect.

An alternative use of biophysical methods in primary screening is in testing a much smaller library of low molecular weight compounds in so-called fragment-based screening [17]. This was first described employing NMR screening to identify fragments that could subsequently be linked and optimised to make higher affinity compounds [4]. More recently, fragment screening has been described by other biophysical methods including both SPR [18] and TSA [19].

### High-throughput screening – affinity selection mass spectrometry (AS-MS)

The application of mass spectrometry (MS) in the drug discovery process has been well established for many years. It has been applied both in the characterisation of target proteins, where it has been primarily focussed on quality control as well as target identification and validation. For small molecules, the technique has been used for metabolism and pharmacokinetics studies as compound identification. More recently, MS approaches have been developed to detect and characterise protein-small molecule complexes.

Affinity selection mass spectrometry (AS-MS) is now the predominant biophysical method used for primary screening. It was first introduced in the late 1990s [20] and various formats have been described, evaluated and implemented since then [21]. However, the basic premise of all these methods is the detection of compounds that bind to the target protein using mass detection of the bound ligand. This simple binding assay has the benefit of reduced interference, since there is no requirement for functional activity which often entails more complex assays and therefore often increased probability of artefacts arising due to effects on the read-out, rather than from true target engagement. Another advantage is that this screening approach can be applied to orphan genomic targets and targets for which functional assays cannot be developed. The major disadvantage, alongside the requirement, mentioned above, for assessing functional activity post-screen, is that these methods are not commercially available and systems, expertise and sometimes even the software solutions required for data analysis have to be developed in house.

**Figure 2.**
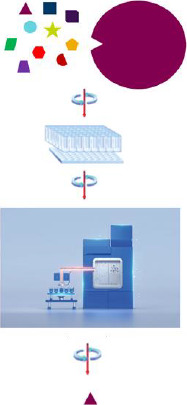
Schematic representation of the workflow for AS-MS primary screening. Mixtures of test compound are incubated with the target protein before separation of bound ligands from free ligands by size plate-based exclusion chromatography. Bound ligands, which are eluted with the target protein, are subsequently detected by mass spectrometry.

Although there are subtle differences in the way AS-MS methods may be employed, the principle of the different approaches is essentially the same. It involves incubating a library of small molecule compound mixtures with the target protein, separating the bound small molecules from non-binders and detecting those binders using mass spectrometry, Figure 2.

The relatively slow MS detection still limits the throughput of these methods and even with the development of acoustic mist ionisation approaches [22], which have increased the speed of mass spectrometry detection, large compound mixtures are still required with mixture sizes ranging from around 100 to almost 3000 compound per well. Consideration of the composition of these mixtures is important as these mixtures have potential for introducing solubility issues, compound-compound reactivity and instability. Smaller mixtures are clearly less disposed to these types of problems but may impose restrictions on the size of the compound library that be screened. Whilst this method is not dependent upon protein function, MS has a low tolerance to detergents present in biological buffers. This is primarily due to the propensity for large aggregates to form which may interfere with binding and detection. Thus, the application is limited to screening soluble proteins as the detergents required for membrane protein preparations are not compatible.

Even with these limitations, AS-MS still represents an important addition to the high-throughput screening toolbox. The sensitivity means that relatively low amounts of target protein are required, of course there is no requirement for labelling of reagents and the process can be automated, all features that contribute to the quality, speed and cost considerations required when deciding upon a primary screening approach.

### High-throughput screening – thermal shift assay (TSA)

The thermal shift assay (TSA) also known as Thermofluor or differential scanning fluorimetry has been used for several years to study protein stability [23]. It is a rapid and simple method that allows the melting temperature, *T*_m_ (temperature at which 50 % of the protein is unfolded), to be determined under different conditions. It has extensively been used to optimise buffer conditions for X-ray crystallographic studies on soluble proteins, such that those conditions yielding the highest stability may be used as start points for crystallisation trials. The addition of compounds that bind to the target protein, thermodynamically stabilise the protein relative to controls in the absence of ligand, and this stabilisation can be used to identify binders from non-binders.

**Figure 3.**
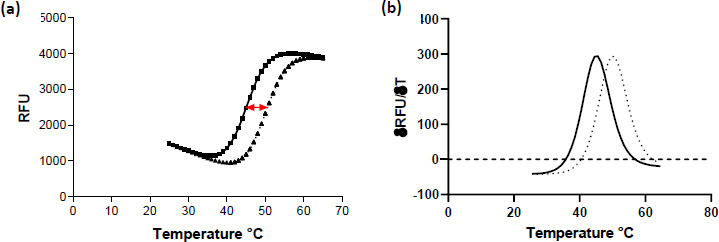
**(a)** Typical unfolding curve in a TSA. Squares / solid line show the protein unfolding in the absence of ligand. Triangles / dotted line show unfolding in the presence of a compound that stabilises by 5 °C. The red arrow indicates the shift in *T*_m_ caused by the addition of compound; **(b)** First derivative of the data in (a).

The TSA experiment involves incubating the target protein with test compounds in the presence of a dye that binds to hydrophobic regions of the protein. The temperature is then increased uniformly, and the fluorescence of dye monitored with temperature. As the protein unfolds more hydrophobic regions are exposed, there is increased dye binding and the fluorescence intensity increases. Compounds that increase the thermal stability of the protein may be identified as those giving an increased *T*_m_, resulting from a shift in the unfolding curve to higher temperatures, Figure 3(a).

The advantages of TSA as a primary screening method include the simplicity of the approach, the cost effectiveness and the potential to access a wide range of binding affinities. The method requires little assay development, which is in effect limited to adjusting [protein] and [dye] to give a suitable signal. The reagent requirements are protein and dye only, meaning that the costs associated with expensive biochemical reagents are avoided. The method does not require specialised instrumentation and is carried out using standard thermocyclers often used for real-time polymerase chain reactions (RT-PCR). Protein quantities can potentially limit the method, since screening 500,000 single wells can require around 0.5 g of a 40 kDa target protein. Whilst there is no direct correlation between Δ*T*_m_ and *K*_d_ [24], due to differences in enthalpic and entropic contributions to binding affinity having a differing effect on Δ*T*_m_, it is possible to observe stabilisation conferred by ligands covering a wide range of affinities from mM to very tight-binding.

Analysis of TSA data typically involves determining the *T*_m_ for each curve, which can be achieved by fitting an appropriate equation to the data, or for simple 2 state transitions, simply by taking the first derivative of the fluorescence versus temperature data, Figure 3 (b). For high-throughput analysis Genedata Screener^®^ (Genedata, Basel, Switzerland) includes a module that can robustly and efficiently analyse the many temperature curves created, which significantly reduces the analysis time.

### Fragment-based screening

Fragment-based drug discovery (FBDD) is now a well-established approach with FBDD having delivered 2 marketed medicines (Vemurafenib and Venetoclax) and around 35 compounds currently in clinical trials [25]. Identifying fragment hits is a challenge in traditional biochemical assays, since they are likely to bind weakly to the target protein, and the apparent affinity may be weaker still, due to competition with substrates. Biophysical methods are well suited to fragment-based screening [25], as they are sufficiently sensitive to detect weak interactions, and reliable enough to avoid false hits. Historically, techniques including NMR, SPR and X-ray crystallography were applied to fragment screening, but more recently methods such as microscale thermophoresis (MST) [26] and TSA have also been used.

Compared to traditional HTS, the throughput of fragment screening is generally lower, and the biophysical methods employed often require larger quantities of protein. Fortunately, this issue is overcome by the ability to screen fewer molecules, but to cover a much larger proportion of chemical space using fragment screening libraries. For example, screening 1 million compounds out of the estimated 10^30^ compounds that could potentially be synthesised with 36 heavy atoms (around 500 Da) is 10^19^-fold less efficient than screening 1000 compounds from the 10^8^ potential compounds with 12 heavy atoms (around 160 Da).

Another way of considering this is to reframe the coverage of chemical space by considering how many compounds need to be screened in order to obtain a sufficient number of hits against the target of interest. Hann, et al [27] found that hit rates decreased as the complexity of ligands increased. Of course, smaller ligands will generally bind less tightly and so the apparent hit rate depends upon the sensitivity of the detection method. Thus, the probability of detecting binding for ligands of different sizes is expected to be low for very small ligands (due to detection sensitivity) high for small ligands and decreases with size for larger ligands (due to the increasing probability of steric clashes between the ligand and protein). This suggests that focusing on fragments with lower heavy atom counts with the most sensitive biophysical screening methods provides the highest probability of success.

NMR is well-suited for fragment screening, as it can detect binding for fragments having millimolar *K*_d_ values. Two general approaches may be applied for proton NMR-based screening, which monitor either differences in the spectra of the small molecules or the protein.

Ligand-based screening methods are often used for medium-sized proteins but work better with larger target proteins. No isotope labelling is needed, and the quantity of protein required is relatively small. Another advantage is that when ligand-observed screens are undertaken, knowledge of the chemical shift pattern for each ligand avoids the necessity for deconvolution. Disadvantages of direct-detection ligand-based screens are that ligand-based screening does not provide information on the binding site, and often false positive rates may be greater than with protein-observed methods, as it is sometimes difficult to discriminate between promiscuous, non-specific binding due to compound aggregation and site-specific binding. Finally, and almost uniquely for biophysical methods, ligand-based NMR screening becomes challenging when binding is too tight. These issues can be overcome by using a reporter or “spy-molecule” in the NMR experiment. However, this requires that such a ligand is available when the assay is developed and has the disadvantage that compounds binding at a non-overlapping site may be missed.

Probably the most robust fragment screening method is protein-observed NMR, where changes in chemical shift for an isotopically labelled protein are monitored. The advantage of using this method is that not only can hits be detected, but affinities can be determined, and binding sites identified if the protein signals have been assigned. This method is suitable for proteins of around 10 – 50 kDa providing that uniformly ^15^N-labeled protein can be obtained. A detailed description of the application of different ligand and protein observed NMR methods in fragment-based screening is beyond the scope of this article, but a valuable overview is given by Harner et al [28].

Since fragments usually have weak binding affinities, they are almost always in the fast exchange regime, and *K*_d_ values can be calculated from changes in chemical shifts with increasing fragment concentration. However, if fragment binding affinity is higher or fragment optimisation leads to compounds that have improved affinity (for example *K*_d_s of the order of 10 - 50 μM) then intermediate exchange of resonances can become a problem, with resonances broadening and disappearing and NMR is no longer useful for *K*_d_ determinations. At this point, other biophysical techniques such as ITC and SPR are often used to measure affinity.

SPR can also be applied to primary fragment screening, and many of the technical practical challenges including working with low-molecular weights compounds with limited solubility and showing low-affinity interactions in high refractive index solvents such as DMSO have been addressed through appropriate assay design and control experiments. Improvements in instrumentation and data analysis procedures have also helped to position SPR alongside NMR in the consideration of methods for delivering fragment screening hits [29].

Immobilisation of the target protein in a functional manner is still a key factor, and several different strategies may need to be explored. Our experience suggests that use of an Avitag^TM^ (Avidity LLC) [30] with coupling via biotinylation of the tagged protein and capture on a streptavidin chip often produces a suitable surface for fragment screening, and subsequent characterisation. Issues with potential fragment binding to the streptavidin are usually overcome by blocking with suitable biotin analogues. Other tagging approaches for immobilisation have been used successfully to capture membrane proteins, providing the possibility of using FBDD versus members of this important class of drug targets, such as G-protein coupled receptors (GPCRs) [31].

As mentioned previously, fragment screening using TSA has also proved to be a useful approach, and has been exemplified as a primary fragment screen, with detailed biophysical follow up, for identifying fragment hits with the potential for disrupting protein-protein interactions (PPIs) [32]. This method, whilst not being suitable for membrane proteins, has the advantage of not requiring immobilisation or labelling of the protein, and so may be more widely applicable to proteins that may be difficult to work with in NMR or SPR.

Microscale thermophoresis (MST) [33] is a developing technique that has also been applied for fragment screening [34]. MST detects the movement of fluorescent molecules in a microscopic temperature gradient created by focusing an infra-red laser beam on a section of a microliter-volume capillary. Binding of ligands typically changes the size, charge, and/or hydration shell of the target protein, producing a change in the thermophoretic movement of the protein. MST requires that the position of one binding partner can be fluorescently monitored, so for screening purposes this is most likely the target protein. Whilst this can potentially introduce artefacts, and the low protein requirement the absence of a need for immobilisation can position this method as useful approach if NMR or SPR cannot be used.

**Figure 4.**
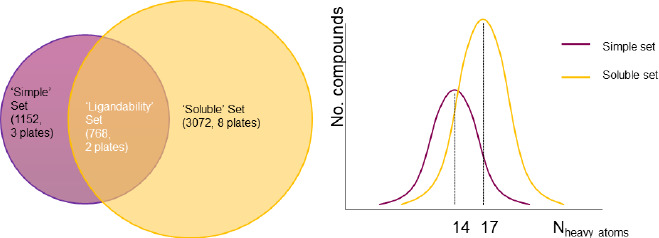
Description of the AstraZeneca core fragment library.

Fragment screening is clearly amenable to a range of biophysical methods, due in large part to the reduced numbers of compounds that are routinely tested. At AstraZeneca the fragment library consists of a soluble set of around 14000 compounds. Within that is contained a core set of 3456 compounds which are routinely used for biophysical screening. This library is further divided into a soluble set used for SPR screening of 3072 compounds and a simple set of 1152 compounds, which are often screened by NMR. There are 768 compounds that are common to both the soluble and simple sets. The design and usage principles behind these sets were that all compounds should have a heavy atom count of less than 20, have molecular weight less than 275, a calculated log *P* of less than 3 and have a predicted aqueous solubility of greater than 100 μM. Additionally, the soluble set was designed for SPR screening, so contained compounds that were larger (potentially important for the indirect mass detection), more complex, and had previously been checked for issues of aggregation / interaction with the dextran matrix by ‘clean-screening’ [35]. The overlapping 768 compounds formed a ‘ligandability’ set that were of intermediate complexity, soluble and ’clean’ and could be used to assess ligandability by any appropriate biophysical method, Figure 4. Using these sets of compounds enables an efficient workflow for both initial ligandability assessment and primary fragment screening by NMR and SPR.

## Hit confirmation

**Figure 5.**
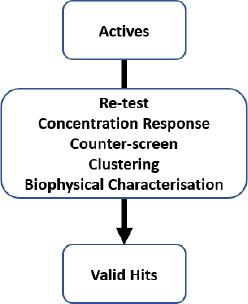
The combination of approaches that are used to triage from primary screening actives to validated hits.

To ensure that hits identified via HTS are valid before subsequent time and resource are invested in optimisation activities several steps are applied in the workflow shown in Figure 5. This involves re-testing actives in the same assay format as the primary screen to confirm that the activity is reproducible. Concentration response assays are then employed to measure the concentration of test compound that brings about 50 % of the maximal effect (EC_50_). A range of counter screens may be used to determine whether potential hit compounds demonstrate artefactual behaviour, if they possess unwanted mechanisms of action or whether they also affect a number of related targets whose modulation should be avoided for selectivity reasons. Typically, compounds are then grouped into clusters, representing the active compounds from each interesting chemical series. This allows a small number of representative compounds from each cluster to be tested in a suitable biophysical technique. Positioning biophysical testing early in this way allows focus on those compounds that are confirmed by a biophysical method and de-prioritising those that may likely be false positives. In this context, biophysical methods provide a means of selecting compounds based on positive selection criteria such as target engagement, mode of action, and for some methods even functional activity (MS, NMR and even ITC can for instance, be used to monitor inhibition of substrate conversion for enzymatic reactions), rather than the negative criteria associated with filtering compounds for unwanted mechanism of action.

As with other application areas of biophysical methods, each has its own strengths and weaknesses in hit confirmation and the choice of method will depend upon the throughput required, the amount of protein available, the affinity range expected and the information content desired, Table 4. If possible, the hit confirmation strategy may include several techniques to provide increased confidence and deliver a deeper understanding of the binding interaction. Positioning one or more biophysical methods will depend upon what is known about the target protein, what is required in terms of setting up each biophysical method and the information desired.

An important consideration is how biophysics might be used in the confirmation of hits resulting from cell-based screening. Of course, for isolated protein-based biochemical assays, the same or similar protein constructs may be used for the biophysical triage step, and so the physiological relevance (or lack thereof) of each is at least consistent. This is different for the case of cell screens, where the biophysical approach may be considered less physiologically relevant than the cell assay. Biophysical methods still have value in confirming binding and providing additional information, but extra caution needs to be applied in comparing the results and in making decisions about fate of compounds based on similarities or differences. In this situation, there is additional value in the use of tool compounds which may have similar modes of action to HTS hits. Confidence can be gained for those HTS hits that show similar behaviour to the tool compounds during the biophysics confirmation stage. It may also be prudent to explore additional (biophysical) methods that allow interrogation of target engagement in cells such as CETSA (cellular thermal shift assay) [36].

## Mechanistic characterisation

Biophysical methods are extremely valuable in helping to carry out in depth characterisation of protein-ligand binding interactions. They often provide a simpler way of complementing biochemical approaches in providing kinetic, thermodynamic and mode of action information. For example, kinetic binding information can be obtained directly using SPR, whereas traditional enzyme kinetics experiments are required to access rate constants for slow-binding interactions. This often entails establishing time-courses under suitable concentrations of substrate(s) for which the control is linear and then observing the slow decrease in enzymatic rate as the test compound equilibrates with the target protein. These types of experiments are time-consuming and often can be difficult to analyse to extract the relevant rate constants controlling ligand binding. A further example is discerning order of addition of substrates to the enzyme in the reaction mechanism. This can be assessed using traditional enzyme kinetic experiments where the rate is measured whilst varying one substrate concentration in the presence of a fixed concentration of the other substrate. This can also be extended to test compounds. However, it can be complemented relatively straightforwardly by using ITC to determine whether the presence of one ligand (for example substrate) is required or competes with binding of another (a second substrate, or analogue, or a test compound).

ITC can also provide the values of the thermodynamic contributions (enthalpy, Δ*H* and entropy, Δ*S*) to the binding affinity directly, and from the temperature dependence of the *K*_d_ values, van’t Hoff values can be calculated from any of the other biophysical methods. The same temperature dependent studies for the association and dissociation rate constants measured by SPR lead to transition state energies for the association and dissociation steps of the binding reaction which enables construction of detailed thermodynamic reaction pathway models for protein-ligand binding. These approaches were used to provide structural and dynamic insights into the binding of different compounds to FGFR1 kinase, to understand the energetics required for movement of the activation loop [37]. Although, it can be difficult to predict these values or to use this type of thermodynamic data directly to design new compounds during lead optimisation, having the ability to dissect the contributions to binding and transition state free energies can provide valuable understanding that will ultimately lead to a more thorough appreciation of the features that contribute to high affinity binding interactions.

More interest, perhaps rightly, seems to have been focussed on kinetic parameters compared to thermodynamic data and the value of combining knowledge of target-compound residence times with pharmacokinetic (PK) and pharmacodynamic (PD) data seems to be gaining awareness [38]. The ability to combine kinetic data from SPR on isolated proteins with data from cell washout experiments provides an extra level of information during lead optimisation. For example, the utility of PK/PD modelling, which attempts to describe the kinetics of the effects of compound following administration, is likely to be enhanced by direct measurements of the kinetics of compound binding and target turnover.

## Case study 1

### Biophysical characterisation of PHGDH fragment hits

**Scheme 1:**
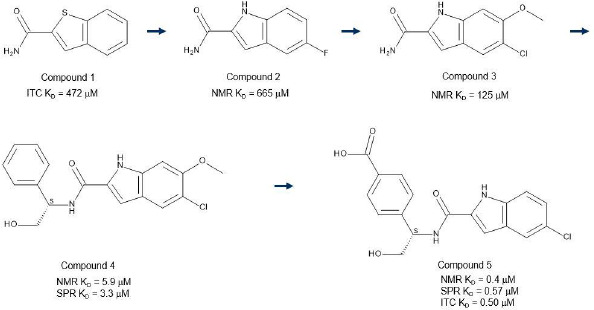
Initially identified fragment binder (compound 1) and key compounds (2 – 5) that were made in the search for a cell active compound to probe the biology associated with PHGDH as a drug target.

**Figure 6:**
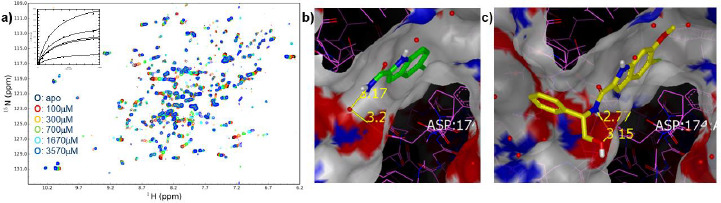
**(a)** 1H15N-TROSY- HSQC spectra illustrating binding of compound 2, compound concentrations used are shown as is the plot of the chemical shift change versus compound concentration used to determine the affinity (*K*_d_ = 980 ± 60 μM); **(b)** crystal structure of compound 2 bound at a resolution of 1.95 Å in the adenine pocket of the NAD binding site showing a water-bridged interaction from the carboxamide to Asp-174 **(c)** crystal structure of compound 4 shows displacement of the water mediated interaction with Asp-174 and opportunities to add additional functionality on the phenyl ring of the benzyl amide, which led to the synthesis of compound 5.

The enzyme 3-phosphohydroxyglycerate dehydrogenase (PHGDH) utilises oxidised ninotinamide dinucleotide (NAD+) and catalyses the conversion of 3-phosphohydroxyglycerate to 3-phosphohydroxypyruvate in humans. The conversion of 3-phosphohydroxyglycerate to 3-phosphohydroxypyruvate is the first, and rate-limiting step, in synthesis of the amino acid serine. Studies have linked PHGDH to the in vivo tumourigenesis in aggressive breast tumours and functional genomics reveal that the serine synthesis pathway is essential in breast cancer [39]. Thus, the druggability of the target was explored with small molecules. To this end a fragment screen of 384 fragments was undertaken using crystal cocktail soaking (mixtures of 4) against the NAD binding domain of PHGDH and 34 hits were identified. The binding affinities of these hits were determined in a 2D NMR binding assay using 15N-labeled NAD binding domain, Figure 6a. These data, in conjunction with X-ray crystallography data, Figure 6b, were used to identify fragments with the greatest potential for development, Scheme 1. A simple analogue of an initial crystallography hit (compound 1), the 5-fluoroindole-2-carboxamide (compound 2) bound at a resolution of 1.95 Å in the adenine pocket of the NAD binding site (Figure 6b). The indole NH forms a water-bridged hydrogen bond network with Ser-211, and the 5-fluoro substituent fits nicely into a lipophilic cleft in the binding pocket. In addition, the carboxamide of compound 2 makes a water-bridged interaction with Asp-174 and the crystal structure shows favourable vectors to grow the fragment into the phosphate binding site. In this endeavour, compounds were made that should displace the water mediating the hydrogen bond interaction between the carboxamide of compound 2 and Asp-174. The synthesis of the α-hydroxymethylbenzylamide, compound 4, led to improved affinity to the single digit μM. A crystal structure of compound 4 (Figure 6c) showed clear opportunities to further explore the phosphate binding pocket by installing additional functionality on the phenyl ring of the benzyl amide and this led to the synthesis of compound 5 with a further 10-fold improvement in affinity.

**Figure 7:**
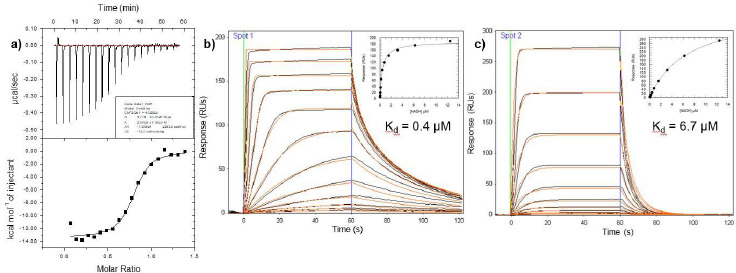
**(a)** ITC titration data for the binding of NADH to full length PHGDH, *K*_d_ = 0.33 ± 0.08 μM, N = 0.78 ± 0.01. **(b)** SPR titration data for NADH binding to full length PHGDH, *K*_d_ = 0.4 ± 0.03 μM determined from a steady state fit, with the kinetic fit curve shown in the inset. **(c)** SPR titration data for NADH binding to NAD binding domain of PHGDH, *K*_d_ = 6.7 ± 0.5 μM determined from a steady state fit, with the kinetic fit curve shown in the inset.

Despite the abundant 2D NMR and crystallography data clearly demonstrating that the NAD binding domain of PHGDH was a folded and stable protein capable of binding ligands, further biophysical data comparing the function of this domain with the full-length protein highlighted differences. A combination of ITC and SPR measurements, Figure 7a-c, clearly demonstrate that NADH binds approximately 20-fold more tightly to the full-length protein. Considering this knowledge, a 1D NMR competition assay with NAD+ as the reference compound was developed. This screen employed the more physiologically relevant full length PHGDH protein, to provide continuity for affinity measurement during the subsequent analoguing phase. It is noteworthy that fragments and many of the synthesised analogues had no measurable activity in the human PHGDH protein NAD fluorescence intensity biochemical assay. Whilst the biophysical methods allowed a wide dynamic range of weak mM to sub μM affinities be determined, the biochemical assay could only be used to provide affinities that could drive SAR once those affinities were sub micromolar. In summary, a fragment crystal cocktail screen, supported with biophysical affinity measurements, provided the first known small molecule known nanomolar inhibitors of PHGDH and valuable tools for interrogating the biology of this target.

## Case study 2

### Biophysical characterisation of hits from DNA Encoded Library (DEL) screening

**Figure 8.**
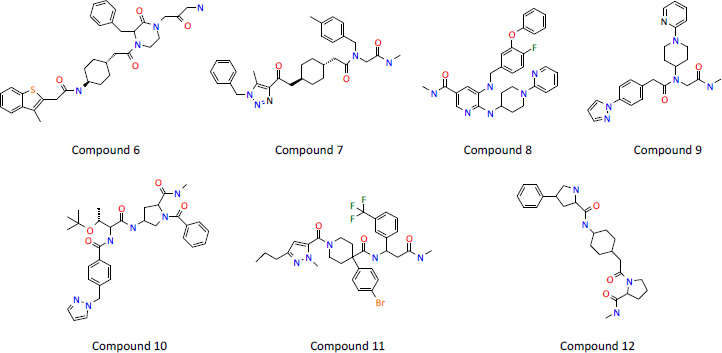
Compound structures identified via DEL screening and profiled using SPR.

DNA-encoded library (DEL) technologies [40] offer an alternative to traditional high-throughput screening and have the unique ability to interrogate very large compound libraries (of around 10^9^–10^11^ small molecules). Whereas traditional HTS methods often rely on activity-based isolated protein or cell-based assays, the DEL screening method is based on affinity selection versus an immobilised protein target, is rapid and requires only microgram quantities of protein. Targets can be screened under multiple different experimental conditions in parallel. For example, different protein concentrations, addition of different cofactors or competing inhibitors, including proteins which represent important selectivity targets could all represent selection conditions that represent useful ways for identifying novel start points, with potentially differentiated modes of action. In a DEL screen versus the acyl carrier protein enoyl reductase InhA, a pool of 11 DNA-encoded libraries consisting of more than 65 billion on-DNA compounds were tested and subsequent selections made utilising different forms of InhA including the apo protein, InhA.NAD^+^, and InhA.NADH complexes. Analysis of output highlighted four general profiles of hits: (i) enriched only versus apo InhA, (ii) enriched only versus InhA.NAD^+^ complex, (iii) enriched only versus InhA: NADH complex but not in the presence of an added inhibitory small molecule, and (iv) enriched in the presence of InhA.NAD^+^ and InhA.NADH but not in the presence of an added inhibitory small molecule. Compounds, which were subsequently synthesised off-DNA were tested in in vitro enzyme assays as well as profiled biophysically using SPR. Compounds were injected either alone, in the presence of 2 mM NAD^+^, or in the presence of 100 μM NADH to monitor binding to different forms of the protein. Where required, excess cofactor was included in the compound injections to ensure that the protein remained saturated with cofactor. Compounds selected from these different conditions could then be easily profiled against apo, NADH or NAD^+^-bound forms of InhA and their respective affinities determined from SPR experiments, Table 5, allowing potentially differentiated profiles to be investigated. The DEL technology allowed the identification of multiple classes of InhA inhibitors, some of which had cell-based activity directly from the primary screen. Compounds were identified as cofactor-specific binders of InhA with often with higher affinity for the NADH bound form. Compounds similar to compound 7, Figure 8, were demonstrated to inhibit bacterial growth in *Mycobacterium tuberculosis* minimum inhibitory concentration (MIC) assays and to kill *Mycobacterium tuberculosis* infected human THP-1 cells.

## Discussion

There are a range of biophysical methods that are available for application at many points during early drug discovery. Each method has its own advantages and disadvantages that lead to different applications dependent upon the reagents available and the information content desired. For example, ASMS is most suited to higher throughput screening, whereas SPR is positioned to deliver kinetic data. NMR can provide structural insights and is the preferred approach for primary fragment screening and ITC provides a rapid thermodynamic characterisation of the binding event. The decision about which method or combination of methods to employ can be complex and subject to change depending upon particular projects, even if they are following similar hit identification and lead generation processes. The choice should ultimate focus on what information is required, the numbers of compounds required from which data needs to be generated to provide this information and the timescale required to deliver this data. Of course, consideration of the target itself and the nature of the binding site(s) being targeted may also influence this decision. Ultimately, practical limitations around protein availability, and costs will also need to be taken into account. However, projects often benefit from the impact that a combination of biophysical and biochemical assay derived information can provide, and biophysical methods should be an integral part of any protein centric drug discovery project.

## Conclusions

Biophysical methods have evolved from being employed to address isolated issues with tool compounds or target proteins, experienced in some early stage projects [41], to now being an essential and integral part of the workflow positioned to establish and pursue hit identification and characterisation across the whole portfolio. Over the last 5 years or so, this progression has been driven by the impact these methods have had, an increased throughput for some methods, and the recognition that a better understanding of the reagents, tools, assays and that mechanistic characterisation and differentiation of hits yields a more efficient early stage drug discovery process. This has led to a more focussed use of biophysics alongside the more traditional approaches, such as enzyme and cell-based assays, which has increased the quality of these early hit-finding assays. Biophysical methods also are increasingly used as primary hit finding approaches, and no longer just for small, fragment-based screens, but also for screening increasingly large compound libraries. The recognition that compound binding, can be placed ahead of activity-based screening, in an orthogonal dyad has been embraced by screening groups and it may be especially useful for novel targets, whose functional activity is unknown or difficult to assay. As a result, biophysical methods will remain a key facet for increasing drug discovery success.

## Figures and Tables

**Table 1.** Potential biochemical and biophysical approaches for protein quality control checks.

Group	Methods (biochemical and biophysical)	Example information required for acceptable quality control
**1. Identity**		
	Amino acid analysis & sequencing	Exact, correct sequence identified
	LC-MS (liquid chromatography-mass spectrometry)	Correct relative molecular mass (M_r_) within instrument error
	Peptide mapping to identify post translational modifications (PTMs) (eg phosphorylation)	Number & sites of phosphorylation; extent of phosphorylation
**2. Purity**		
	SDS-PAGE (sodium dodecyl sulfate – polyacrylamide electrophoresis) / native PAGE	Single band on a gel; still a single band at high loading
	Dynamic laser light scattering (DLS)	Monodisperse, *M*_r_ ± 20 % expected
	Analytical gel filtration	Defined single Gaussian peak for a monomer
	Analytical ultra centrifugation (AUC)	Indicates homogeneity & correct *M*_r_
**3. Concentration**		
	Ultraviolet (UV) spectrum	Peak at 280 nm; Peak at 205 nm; No peaks above ~ 340 nm; Test for light scattering (look into ratio at different wavelengths eg A280/A230); concentration calculated using ε
	Bradford assay	Linearity with BSA standards
**4. Functionality**		
	Functional assay	Functional activity observed with expected parameters (eg *k*_cat_, *K*_m_, *k*_cat_/*K*_m_)
	Isothermal calorimetry (ITC)	With known tool ligand: n ± 15 % of expected; *K*_d_ within 2-fold of reference value; ΔH within 1 kcal/Mol
	Surface plasmon resonance (SPR)	Direct binding assay (DBA): *K*_d_ within 2-fold of reference value; Expected theoretical *R*_max_; Inhibition in solution assay (ISA): [Protein] within ±15 % of two different concentration measures (Bradford & A280); competition observed between target definition compound (TDC) and TDC in solution
	Functional comparison between protein batches	Compare K_d_,ΔH, stoichiometry, *K*_m_, *k*_cat_, *k*_cat_/*K*_m_ (usually > 10^6^ s^-1^ M^-1^), *K*_i_; Single phase kinetics
	Validity of construct	Compare *K*_d_, *K*_m_, *K*_i_,Δ*H* with full-length protein; compare structure-activity relationship (SAR)
**5. Stability**		
	Differential scanning calorimetry (DSC)	Good pre-transition baseline; visible Tm (above 37 °C); good post-transition baseline
	Differential scanning fluorimetry (DSF)	Good pre-transition baseline; visible Tm (above 37 °C); good post-transition baseline
	Selwyn’s test	Overlay of plots of [P] vs [E].t for different combinations of [E] and t

Where LC-MS is Liquid chromatography mass spectrometry, PTM is post translational modification, UV is ultraviolet, Mr is relative molecular mass, BSA is bovine serum albumin, ε is the molar extinction coefficient, *k*_cat_ is turnover number, *K*_m_ is Michaelis constant, n is stoichiometry, *K*_d_ is equilibrium dissociation constant, Δ*H* is binding enthalpy, DBA is direct binding assay, ISA is inhibition in solution assay, *R*_max_ is maximum response, TDC is target definition compound, SAR is structure activity relationship, *T*_m_ is the the melting temperature, [P] is product concentration, [E] is enzyme concentration, *t* is time.

**Table 2.** Examples of factors that have compromised protein integrity for use in drug discovery projects, resulting observations and actions taken to overcome the issues.

Protein target	Quality control issue	Biophysical methods employed	Observations	Actions taken
Lactate dehydrogenase	Cofactor present in protein preparation	ITC, SPR	Tool compounds and added cofactor binding more weakly than expected	New purification method established
ATAD2	Protein aggregation	NMR, ITC, TSA	Protein showing poor spectrum, negative shifts with compounds in TSA, no binding of tool compounds	New construct designed
ACPER	Reduced binding functionality	ITC	Low stoichiometry and enthalpy for cofactor binding	New batch of protein prepared
MAPKAPK2	No binding to p38	NMR, ITC	Short construct used for NMR did not show binding to p38 and differences in compound affinity observed for long and short constructs in phosphorylation assays	Longer construct, containing putative site for p38a binding, used for activity and mechanistic assays

Where ATAD2 is ATPase family AAA domain-containing protein 2, ACPER is acyl carrier protein enoyl reductase, MAPKAPK2 is MAPK activated protein kinase 2.

**Table 3.** Impact of biophysical evaluation of hits identified during HTS development.

Protein target	Assay methodology	Number of compounds tested	Biophysical methods employed	Observations	Actions taken
KEAP1	HTRF	180	NMR, SPR	No genuine hits identified	HTS was stopped and FBLG approach used instead
MALT1	Fluorescence intensity following proteolytic cleavage	60	NMR	17% of hits showed specific binding. 38% showed redox cycling behaviour. 27% were not soluble	Incorporation of a redox-artefact assay in the cascade reduced the number of redox-active compounds reaching the NMR assay from 38% to 5%
ERRγ	FRET	180	NMR	FRET assay suggested that hit rate would be low. NMR suggested that 90% were false positives	HTS in this format was not run
TTBK1	ADP-glo	250	SPR	69 verified hits, then profiled versus phosphorylated and non-phosphorylated protein Large number of reactive compounds identified	ADP-glo assay was not run
ACPER	Fluorescence intensity following substrate turnover	Tool compounds and 630 fragments	SPR, ITC	Characterisation demonstrated that compounds showing several different mechanisms of inhibition could be found	Project view on needing a cofactor competitive inhibitor was changed and assays configured to find all mechanisms
LTC4S	HTRF, RapidFire	Total of 50 selected from actives in one or both assays	NMR, SPR	77% of the total hits shown to bind and also to displace tool ligand. Confirmation rate was 90% for RapidFire hits, 40% for HTRF hits	RapidFire assay prioritised for full HTS
aPC	4 different assays: 1) Chromogenic cleavage 2) Peptide cleavage coupled assay 3) Peptide cleavage RapidFire 4) Fibrin clot assay	Total of 250 identified from the assays as follows: 1) 90 2) 50 3) 10 4) 100 190 selected for biophysical testing (90 from assays 1-3 and total output from 4	SPR, NMR	Numbers of confirmed hits originating from each assay approach: 1) 8 2) 16 3) 9 4) 13 Fibrin clot assay was shown to identify compounds binding at a site distal from the active site	Fibrin clot assay was selected for HTS, based on the ability to identify novel, exo site binders.

Where KEAP1 is Kelch Like ECH Associated Protein 1, MALT1 is Mucosa-associated lymphoid tissue lymphoma translocation protein 1, ERRγ is Estrogen-related receptor gamma, TTBK1 is Tau tubulin kinase 1, ACPER is acyl carrier protein enoyl reductase, LTC4S is Leukotriene C4 synthase and aPC is activated protein c.

**Table 4.** Comparison of the some of the most common methods used for hit confirmation.

Technique	Specific requirements	Protein consumption	Throughput	Dynamic range	Information content
NMR	^15^N labelling for 2D protein observed NMR	High	Medium	mM - μM	High (binding site)
SPR	Suitable immobilisation	Low	High	mM - pM	High (kinetics)
ITC	Protein and ligand in identical buffer	High	Low	mM - nM	High (thermodynamics)
MST	Fluorescent labelling of one partner	Low	Medium	mM - pM	Medium (affinity)

**Table 5.** Example of SPR profiling of DEL screening hits

Compound	*K*_d_ μM		
	Apo InhA	InhA.NAD^+^	InhA.NADH
6	>100	13.4 ± 4.3	0.3 ± 0.2
7	>100	46.7 ± 11.6	0.09 ± 0.06
8	>100	>100	0.3 ± 0.1
9	>100	>100	36.8 ± 3.3
10	>100	49.0 ± 2.9	0.06 ± 0.03
11	12.4 ± 1.4	0.3 ± 0.1	5.0 ± 1.2
12	>100	>100	6.3 ± 2.6
